# Travelers’ Subjective Well-Being as an Environmental Practice: Do Airport Buildings’ Eco-Design, Brand Engagement, and Brand Experience Matter?

**DOI:** 10.3390/ijerph20020938

**Published:** 2023-01-04

**Authors:** Aly H. Abdel-Gayed, Thowayeb H. Hassan, Ahmed Hassan Abdou, Mostafa A. Abdelmoaty, Mahmoud I. Saleh, Amany E. Salem

**Affiliations:** 1 Social Studies Department, College of Arts, King Faisal University, Al Ahsa 400, Saudi Arabia; 2 Tourism Studies Department, Faculty of Tourism and Hotel Management, Helwan University, Cairo 12612, Egypt; 3 Hotel Studies Department, Faculty of Tourism and Hotels, Mansoura University, Mansoura 35516, Egypt; 4 StatisMed for Statistical Analysis Services, Giza 12573, Egypt; 5 Marketing Department, Graduate School of Management, Saint Petersburg State University, 199004 Saint Petersburg, Russia

**Keywords:** eco-design, green environment, airport, well-being, experience, engagement

## Abstract

The physical environment of airports plays a crucial role in improving travelers’ perceptions and well-being. Adopting a green physical environment may elicit customers’ cognitive and emotional responses and provide a convenient consumption environment. Brand experience and engagement are other important consumer–firm interactions that influence the attributes of the passengers’ well-being. The current study sought to assess the impact of the eco-design of buildings, brand experience and engagement on the well-being of travelers at an international airport in Saudi Arabia. Additionally, the current study investigated the possible effects of eco-design on airport experience and engagement. The results of the structural equation modeling analysis revealed that the eco-design of airport buildings was independently associated with passengers’ well-being and brand engagement, but not with brand experience. Additionally, well-being was significantly predicted by brand engagement and experience. Airport managers are advised to adopt an internal eco-design to help promote passengers’ connection with the brand and improve their well-being, which would eventually be reflected in their behavioral attributes and decision-making.

## 1. Introduction

The aviation industry accounts for a considerable share of greenhouse gas emissions across the world, and efforts have been made to address the increasing impact of aviation on the environment [1,2]. The anticipated growth in the global number of air passengers, particularly in the recovery period after the COVID-19 era [3], is essentially accompanied by negative effects on climate change, due to fossil fuel consumption [4]. Therefore, multiple organizations and research bodies have sought to implement regulatory measures and adopt strict approaches in regard to manufacturing products in a way that aims to protect the environment. This can be ideally attained through creating environmentally-friendly products and measures based on the expectations of the consumers [5]. Concomitantly, it has been shown that product appearance had a significant impact on consumers’ perceptions [6], and the existence of eco-products has been an important factor in promoting the concept of environment-friendly entities [7].

Therefore, eco-design of buildings has frequently been a matter of research in the tourism sector, and the aviation industry is no exception [8]. Implementing an eco-design in an airport is defined as adopting a human-made environment that potentially impacts on the emotions, mental health, behaviors and physical health of occupants within the airport building [9]. Actually, eco-design is a multifaceted concept that integrates environmental attributes with measures of creating sustainable solutions. These measures eventually help satisfy user desires and needs [10]. Recently, airports have begun to seek ways to ensure that passengers have positive perceptions and attitudes when in the airport, feel engaged with the airport and have good psychological well-being in it, in order to actively ensure customer retention and long-term success in a highly-competitive sector [8,11]. Actually, the efficient utilization of eco-design is expected to be associated with positive outcomes, including reduced emotional exhaustion, stress reduction and customer retention, which are all crucial elements of a company’s success [12]. Accumulating investigations into the cognitive and behavioral impacts of the eco-friendly design of buildings showed that a green physical environment had a positive healing effect on negative feelings, depression, distress and anxiety [13].

However, in the context of airports, little is known about the attributes of customer well-being after the implementation of eco-design in airport buildings, such as eco spaces, living plants, green décor and green atmospherics [13,14]. It has previously been shown that adopting an eco-design would help provide a relaxing consumption environment that supports consumers’ perceptions of well-being [12]. In this vein, travelers’ perceptions of subjective well-being is referred to as the extent to which a given brand would positively contribute to enhancing the impact of quality of life of the service provided by the airport [15]. Subjective well-being is related more to self-evaluation of life satisfaction and happiness, rather than being an objective measurement of economic and health aspects and other well-being attributes [16]. Customer well-being may also be connected to brand-related variables, such as engagement with the airport brand and the overall experience of passengers [11]. Experience is a key element in developing positive memories and experience has frequently been cited in studies focusing on experience as a driving force of the market [17]. As with other industries, experience in the tourism sector is referred to as the interaction between the company and the consumer that elicits emotional interactions providing memorable services [18]. From another perspective, brand engagement has been identified as the level of connection and interaction between consumers and a brand. Consumers’ engagement with a brand has evolved as a channel through which a consumer forms a passion for a brand, and develops an individual disposition that builds commitment towards a relationship with the brand [19]. Customer experience and engagement can both serve as important catalysts for high customer satisfaction and better business outcomes [20]. Therefore, an interplay of customer engagement and experience, as well as customers’ well-being, should be a matter of research.

In Saudi Arabia, to the best of our knowledge, no studies have studied the impact of an airports’ eco-design, and brand experience and engagement, on the psychological parameters of travelers. On the national level, and considering the scarcity of knowledge regarding the topic, determinants of psychological attributes are considered important in future plans to improve the quality of services and enhance marketing performance. Therefore, the creation of a green service-scape environment needs to be traced back and linked to brand-related parameters, eventually reflected in travelers’ satisfaction. The present study aimed to assess the impact of the green physical environment of airport buildings on customers’ subjective well-being (as a function of life satisfaction and happiness), as well as brand experience and engagement. Given that travelers would temporarily be subjected to travel experience in airport lounges in specific time periods, the present study focused on the subjective well-being attribute. Additionally, the current investigation explored the role of customers’ experiences and engagement with the brand on their well-being. Finally, we sought to investigate a potential moderating role of brand experience on the relationship between the eco-design of buildings and subjective well-being.

## 2. Literature Review

### 2.1. The Concept of Eco-Design of Airports’ Physical Environment

The physical environment of buildings is a term used interchangeably with building design and building atmospherics [11]. Building design is an important factor that facilitates the buying process by generating distinct emotional effects on consumers to increase the likelihood of their purchasing a product or a service [21]. Airport buildings include the terminals, office building and hangers for the airplanes. The physical environment of traveler terminals has the utmost importance because these are heavily utilized by large numbers of passengers [11,22]. Green design cues include aesthetic and functional elements that affect consumers’ evaluation of a given destination. A biophilic design consists of using natural cues, such as botanical gardens, in order to attract consumers, improving approach behavior and reducing the stressful atmosphere of the daily routine of travelers in airports [23]. It is, therefore, plausible to design airport buildings, particularly passenger terminals, to a passenger-friendly pattern. With the extensive variation of airport designs, greening was adopted as a core parameter in recent designs [24]. Basically, multiple green constituents of airport buildings have been increasingly considered, such as green décor (green items, plants and green walls), green ambiance (natural scents, air freshness and natural light) and green spaces [25].

Notably, eco-designs have been linked to passengers’ emotional responses and self-evaluation of buildings [26]. There is a growing body of evidence indicating the role of environmental psychology and health atmospherics in supporting psychological health and enhanced experiences with services and products, in order to enhance post-purchase behaviors in the tourism and hospitality sectors [23,27]. Additionally, many researchers assessed the factors associated with a green physical environment, such as consumers’ attitudes, resilience, satisfaction, and brand engagement [9,28,29]. For instance, Han and Hyun [9] recently showed that a green environment (in outdoor and indoor settings) played an important role in improving mental health perceptions, loyalty and emotional well-being.

Therefore, the following hypotheses were developed:

**Hypothesis 1 (H1):**  *Eco-design of the airport significantly influences brand engagement.*

**Hypothesis 2 (H2):**  *Eco-design of the airport significantly influences brand experience.*

**Hypothesis 3 (H3):**  *Eco-design of the airport significantly influences travelers’ subjective well-being.*

### 2.2. Brand Experience and Engagement

Designing a green physical environment is an important facet of efforts aimed at airport greening [11]. Indeed, since the physical environment frequently provides a tangible cue that can be relied on [30], it is critical to underline the tangible experience of tourists and visitors when designing tourism products and services [11,27]. Furthermore, the eco-design should be a tangible cue when visitors provide judgements on their experience at the airport. Researchers in previous studies investigated the green physical environment in airports, including green items (walls, plants, etc.), green ambient conditions, and green spaces, as well as resting areas, hallways, waiting lounges and restaurants [5,11,27,31,32]. Based on these studies, there is a consensus that a green physical environment induces positive responses in individuals’ consumption behavior and enhances their experiences. Unsurprisingly, passengers’ experiences, defined as the interactions and activities that the passengers have in an airport [33], elicit emotional connections and excitement about the service and/or product [34]. Interestingly, brand experience involves a cumulative experience of multiple contact points along the consumer journey rather than a single touch point with the brand [35]. Brand experience consists of several domains, including sensory (the experience as encountered with the senses), behavioral (the undertaken actions by consumers due to the experience), affective (related to emotional interactions due to the experience) and intellectual (due to perceptions and thoughts formed as a result of the experience) [36], The current study focused on the sensory evaluation because it is the most influential domain in decision-making [12]. Brand experience at airports includes the link between passenger and airport objects and staff. The active participation of travelers mediates deeper feelings. Discomfort with a given brand would elicit negative feelings, which might eventually impact travel experiences [37]. Considering the fact that the eco-design of a store building mediates a relaxing environment and a well-being consumption paradigm, visitors may form positive behaviors, increase brand engagement and enhance reputation or image of the brand [12]. Collectively, brand experience is influenced by service quality and physical services, and the experience, in turn, impacts brand engagement and well-being.

Of note, brand engagement is another important attribute in the understanding of marketing domains. An engagement with a brand encompasses a number of non-transactional behaviors which are elicited because of the consumers’ interests [38]. It is a multidimensional concept that relies on customers’ expressions of their emotional, cognitive and behavioral attributes [39,40]. Therefore, brand engagement is referred to as the level of the consumer’s state of mind related to the brand, self-motivation and the context, and is characterized by distinct levels of behavioral, emotional and cognitive activities during the interaction with a brand [39]. Few studies have examined the impact of green practices on brand engagement. Lee et al. [41] indicated that a green physical environment at luxury hotels had favorable evaluations compared to hotels with non-biophilic designs. These favorable evaluations included economic value and attitudes, which are antecedent predictors of customer engagement [41,42]. This was corroborated by Alfakhri et al. [43], where green designs in the hospitality industry impacted customer experience and subsequent purchasing behaviors. Chuah et al. [44] showed a significant correlation between perceived corporate social responsibility of airline corporations on sustainable customer engagement, and such a relationship was significantly moderated by green trust and environmental concerns. These findings guide airlines in addressing the effects of corporate social responsibility and green practices on brand engagement and communication [44].

Therefore, brand engagement acts dynamically, where a passenger interacts with the airport across the travel experience [45]. Travelers can promote the airport services, staff, and facilities, and the connection-related measures undertaken by airports can ultimately improve brand engagement [46]. Brand engagement is a common attribute which encourages airports to improve their services so that this is reflected un engagement behaviors [47]. Therefore, brand engagement is another measure of brand equity in the airport industry. Notably, there is a potential interaction between brand engagement and experience. This is because consumers’ experience may be quickly attained, or time may be required to develop engagement with a brand before having a good perception [48]. Therefore, active engagement may mediate a good brand experience for passengers.

### 2.3. Travelers’ Subjective Well-Being

In general, philosophers have defined well-being as the quality of a good life, and others have expanded the concept to a good society [49]. However, more specific terms have been proposed in the subsequently published material. An objective approach of well-being implies that quality of life indicators are the major determinants of subjects’ well-being; these include material resources, such as housing, income and food, as well as social domains, such as health, education, social networks, etc. [16,50]. Another subjective approach has been frequently utilized, which relies on self-evaluation of one’s life. In particular, subjective well-being is mainly oriented towards self-perceptions of life satisfaction (a cognitive attribute) and happiness or unhappiness (an emotional attribute) [49]. There has been a gradual increase in interest in the assessment of subjective well-being, given that it contributes to favorable life outcomes, such that individuals with high levels of subjective well-being possess stronger immune systems, low prevalence of cardiovascular disease and are more pro-social and cooperative [51,52].

In the tourism industry, well-being perception is defined as the perception of travelers of the extent to which a given airport brand positively mediates the quality-of-life enhancement [15]. It relates to self-evaluation of the quality of life within optimal physiological and psychological aspects, and it implies emotional and cognitive evaluation of life [53]. Consumers place importance on enrichment of the quality of life while making purchase decisions. From another perspective, travel is a significant source of positive emotions (e.g., relaxation, pleasure and prestige), and travel can be seen as an important contributor to well-being [54]. Consistent with early research [15,55], a traveler’s well-being was defined as the extent to which a traveler’s experience with a given airline lounge influences that traveler’s self-perceived quality of life. In an airline lounge, the traveler’s experience is perceived to influence the need of well-being if he or she perceives that using the lounge may improve the quality of travel experience.

For example, Liang et al. [56] found that visitors with higher degrees of satisfaction regarding indoor environmental quality in green buildings had significantly higher levels of subjective well-being. Furthermore, Kim et al. [12] assessed the impact of multiple domains, including sensory, emotional and cognitive evaluation, on well-being perception among airway passengers. The results showed that travelers’ cognitive and sensory evaluation of airport lounges were antecedent predictors of well-being perceptions [12]. Cognitive factors relied on items related to physical and non-physical attributes, whereas the sensory factors were primarily focused on service scape attributes that form the immediate responses of travelers [12,57]. In another recent quantitative investigation, Han et al. [58] revealed that specially designated green areas and natural surroundings in an airport exert significant positive impacts on the mental health value of that airport’s occupants.

The travel experience is enhanced when a passenger is relaxed in a comfortable atmosphere or accomplishes what he/she wanted to do [12]. In services marketing, cognitive evaluation of services is known as the perceived quality of services by passengers regarding the overall experience in airline lounges. The interaction between travelers and the facility or service in an airline lounge may also provide cognitive stimulation [12]. Importantly, cognitive evaluation has a significant role in well-being perception [12]. Therefore, passengers’ perceptions of service quality (represented as the eco-design of airport buildings in the current study) may be linked to subjective well-being.

Green ambience also has significant effects on brand image, which indicates that consumers could perceive green ambience favorably [12]. In their study, Han et al. [58] stressed the significant effects of natural surroundings and the green physical environment on an airport’s image. Furthermore, a traveler’s experience in an airline lounge may also influence that traveler’s well-being.

Based on these observations, the hypotheses of the current study were formulated as follow:

**Hypothesis 4 (H4):**  *Airport’s brand engagement significantly influences brand experience.*

**Hypothesis 5 (H5):**  *Airport’s brand engagement significantly influences travelers’ subjective well-being.*

**Hypothesis 6 (H6):**  *Brand experience significantly influences travelers’ subjective well-being.*

**Hypothesis 7 (H7):**  *Brand experience significantly moderates the relationship between eco-design and travelers’ subjective well-being.*

**Figure 1:**
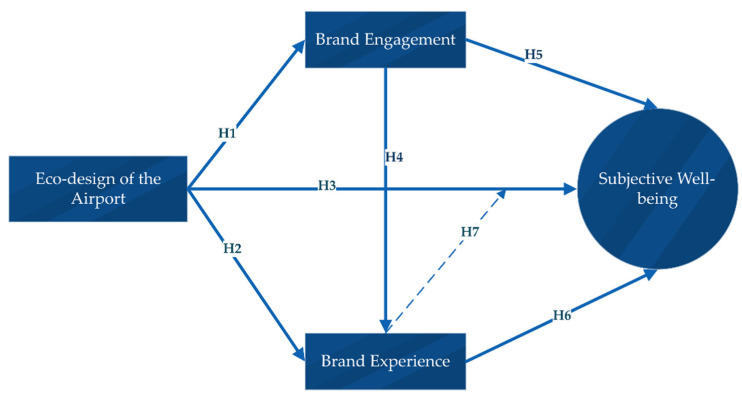
The hypothesized framework of the current study.

A full framework of the hypothesis is illustrated in Figure 1.

## 3. Materials and Methods

### 3.1. Construct Mesures

A survey-based study was conducted adapting questions to cover the holistic idea that served the objectives of the study. Seven items of an airport’s eco-design were adapted from Han et al. [59]. These items showed the dimensions of airport environmental design that travelers encounter during their stays at airports. Moreover, eight items were adapted to evaluate brand management from Prentice et al. [35] and Obilo et al. [38]. These items explored the emotional and rational attachments between passengers and the airports. Additionally, we adapted three items to brand experiences and well-being from Ma et al. [18]. These items helped fathom the essence behind the passenger perception of the airport as a brand and tourists’ behavioral outcomes. These items were collected on a five-point Likert scale, ranging from 1 = Strongly disagree to 5 = Strongly agree. The included items under each domain are listed in the Supplementary Data (Table S1).

### 3.2. Data Collection

Travelling at airports is often a mass phenomenon demanding extensive passenger involvement levels. Thus, the present study collected data using an e-survey, which was chosen since it is easily accessible, cost-effective, and responses are received quickly [60]. We selected the respondents for the current study with a non-probability convenience sample at the King Fahd International Airport. The reason behind this airport being selected, as the context of analysis, is that it is one of the vital international airports in Saudi Arabia and is considered one of the busiest airports in the country [61]. Moreover, we ensured that the participants in the survey had fresh memories of the airports, according to the recommendations of Kim et al. [62]. So, we targeted passengers who had experiences at the airport of not more than one month previously to ensure accurate and specific results. We then distributed the e-survey by informing participants through a multinational travel agency from 01 June to 30 September 2022, at the peak of international passengers being at the King Fahd International Airport in Dammam city, Saudi Arabia.

### 3.3. Statistical Analysis

Data analysis was carried out using RStudio (R version 4.1.1). Categorical variables were presented as frequencies and percentages. Exploratory and confirmatory factor analyses were applied to assess the validity of the proposed model. A partial least squares structural equation modeling (PLS-SEM) approach was used. This method is feasibly used in models consisting of moderating relationships because the indicators are linearly combined to construct composite variables [63,64,65]. The convergent validity was assessed using composite reliability (CR), the exact reliability coefficient (RhoA), average variance extracted (AVE) and Cronbach’s alpha [66,67]. The discriminant validity of the model was assessed by using the Fornell–Larcker (F-L) criteria and heterotrait–monotrait (HTMT) ratio of correlations. The results of the bootstrapped structural path were expressed as beta coefficients (β) and 95% confidence intervals (95%CI).

## 4. Results

### 4.1. Demographic Characteristics

The responses of a total of 352 participants were analyzed in the current study. Females represented approximately two-thirds of the sample (67.9%). More than a half of respondents were married (52.0%) and had obtained a university degree (52.0%). Less than a half of the sample had no children (43.5%). Approximately one-third (34.9%) of the respondents had a monthly income of >12,000 SAR (Table 1).

### 4.2. Internal Consistency and Convergent Validity

To confirm the validity of the survey used, an exploratory factor analysis (EFA) was carried out using a promax rotation. The EFA revealed a model consisting of three constructs. However, one item was excluded from the eco-design of airports domain because it was not significantly loaded to its main construct (factor loading of the variable Eco_07 was 0.49, Table S1). Based on the confirmatory factor analysis, the model showed satisfactory fit statistics (χ^2^ = 358.329, df = 114, *p* < 0.001, CFI = 0.951, TLI = 0.941, RMSEA = 0.078). Furthermore, the standardized factor loadings were ≥0.7, indicating significant loadings (Table 2). The internal consistency of survey subdomains was good, as confirmed by the high Cronbach’s alpha values (ranging between 0.735 and 0.947). Furthermore, the RhoA values exceeded 0.7 and AVE values exceeded 0.5 [68] (Table 2).

### 4.3. Discriminant Validity

To confirm the discriminant validity of our model, the square roots of the AVE values were compared to the correlation between different constructs (Table 3). The results showed that the correlation coefficients were lower than the square roots of AVE. Furthermore, the HTMT values were not higher than 0.85 (Table 4) [69]. In addition, the bootstrap confidence intervals of HTMT were not significantly higher than 1 (Table S2); therefore, the discriminant validity was assured.

### 4.4. Outcomes of the Structural Model

The results of the structural analysis showed that travelers’ well-being was significantly and independently predicted by the eco-design of the airport (β = 0.27, 95%CI, 0.09 to 0.43, *p* = 0.001), brand engagement (β = 0.23, 95%CI, 0.02 to 0.45, *p* = 0.021) and brand experience (β = 0.15, 95%CI, 0.02 to 0.31, *p* = 0.039). The eco-design positively influenced brand engagement (β = 0.82, 95%CI, 0.78 to 0.86, *p* < 0.0001) but not brand experience (*p* = 0.176). Participants’ engagement with the airport brand was also positively influenced their experience with the brand (β = 0.72, 95%CI, 0.58 to 0.87, *p* < 0.0001). However, brand experience had no moderating effect on the relationship between the eco-design and brand experience (*p* = 0.612, Table 5).

## 5. Discussion

### 5.1. Discussion

The results of the current study add to the existing literature regarding the impact of a green physical environment in airports. Based on a robust quantitative analysis, the current study supported the hypothesis (H3) and revealed that the eco-design of airport buildings significantly contributed to enhancing the passengers’ subjective well-being, which is a key concept of success for every business. The well-being was also independently associated with brand experience (H6 was accepted). It was unsurprising that the biophilic building design effectively elicited cognitive and emotional responses, perceived during the overall evaluation of buildings and places in the airport [12]. Practitioners and researchers have stressed that nature provokes health benefits and emotional responses, particularly for individuals who are continually connected to the natural environment [70,71]. The integration of natural elements into a hotel physical environment led to increased customer retention, satisfaction and well-being [72]. Han and co-authors [59] also demonstrated a strong independent relationship between the green physical environment at airports and customers’ subjective well-being. Moon et al. [11] also emphasized the need for a biophilic design to induce affective and cognitive appraisals of passengers’ experiences. These consistent findings stress the importance of green items and spaces in supporting mental health perception and overall image of the brand.

In the present study, we also showed that eco-design was a significant predictor of enhanced brand engagement (H1 was accepted). This was in agreement with previous evidence indicating that employing a green service design in hotels would help in the engagement of customers with the brand [73,74]. Additionally, the eco-design of airport buildings significantly influenced the reputation of airports [59]. The result also agreed with earlier tourism investigations which underlined the importance of brand reputation and engagement in explaining subsequent behaviors and decision-making [75,76]. In the hospitality industry, Lee et al. [77] stated that customers perceive hotels with biophilic designs as being superior in quality compared to those with non-biophilic designs. Another study has similarly shown that customers would have a stronger willingness to visit hotels with a green physical environment and to be engaged with green hotel brands [78]. In a recent study, Rosenbaum et al. [79] studied consumers’ neural activation following exposure to natural elements, and showed that biophilic designs elicited consumers’ interest, attention, and relaxation and supported brand engagement. Firms and marketing entities are becoming aware of the potential benefits of green practices and their relationships with consumer marketplace behavior [80]. Brand engagement is comprised of a two-way interaction path between the consumer and the brand, and the psychological perception of the subjects (consumers) is the most important factor in the creation of engagement. The perceived impacts of a green physical environment affected passengers in a way that promoted their engagement with the brand. Collectively, atmospheric designs have important implications on customers’ attachment and engagement, and this should be exploited in further communicative strategies based on visitors’ familiarity of airports. Incorporating a green environment elicits positive consumer evaluations, supports the well-being construct and enhances the decision-making process.

As mentioned earlier (Section 2.2), a proportion of passengers may need time to become engaged with an airport brand before developing a good brand experience [48]. In the current study, brand engagement significantly impacted the experience; hence, H4 was supported. This was supported by the fact that consumers’ experience formed via a number of stimuli which developed during direct and indirect interaction with a given brand [81]. Basically, brand interaction usually impacted the evaluation process and subsequently influenced post-consumption experiences, attitudes and moods. In this way, brand engagement and consumers’ experience can be linearly correlated [82]. In the current analysis, the eco-design of airports positively influenced brand engagement, and, later, positively affected brand experience. Nevertheless, the eco-design was not associated with brand experience [74]. A possible explanation of this finding is that a single parameter of brand experience was included (sensory experience), and this might have impacted the interactive scheme of the model. However, the findings of the current study showed that eco-design indirectly influenced the sensory brand experience through brand engagement. Ultimately, it seems that enhancing the green environment at airports would support subjective well-being through three pathways, including direct effect and indirect effects, via brand experience and engagement.

### 5.2. Strengths and Limitations

In the current study, a survey with previously validated items was utilized for data collection, and the validation was confirmed statistically on the sample under study. The current investigation employed robust statistical approaches, and the model was well-fit; hence, we could retrieve reliable results. The findings of the current study would fill gaps in the current literature, particularly in the context of scant evidence in the airline industry. Although the impact of eco-designs on consumer behavior have been investigated elsewhere in the tourism literature [83,84,85], little is known about the effects of biophilic designs at airports on visitor behaviors and responses. The results presented in the current study provide a robust foundation regarding the green physical environment of airports in the decision-making process of customer behavior strategies. The current study focused on important attributes that would support the firm’s reputation and consumers’ well-being, which are undoubtedly crucial elements for every business. Particularly, the current study assessed how these attributes could be elicited by utilizing eco-designs, a matter which has scarcely been assessed. Empirically, evidence from the present findings demonstrated the importance of eco-design within the postulated framework, such that it was an essential element that influenced its subsequent constructs in the decision-making process by customers. Consequently, airports should not only stress the functional facets to satisfy passengers’ needs, but also target the emotional and cognitive needs of visitors. In essence, providing a green atmosphere is a fundamental aspect to help visitors feel relaxed, healthy and happy in order to support brand-related attributes and airport reputation when compared to other brands. This could be attained by increasing eco-spaces, living plants, green rest areas and green physical environments. All these elements would eventually support the airport brand, increase the subjective well-being of customers and enhance the behavioral intentions.

However, the study was not without limitations. Data collection was performed based on a convenient sampling approach. Furthermore, the study was carried out among travelers from a single airport. These limitations might limit the generalizability of the obtained results to a greater population in other airports inside and outside Saudi Arabia. Data may also be subject to information bias due to the self-reported questions. Additional studies should involve multiple airports in a single country or in multiple countries, and open-ended questions may be added to the survey to employ a mixed design. A random sampling technique might also be adopted to account for the generalizability options. Focusing on the brand experience domain, the present study exclusively relied on the sensory experience in our study (rather than other behavioral, affective and intellectual domains of experience), and this might have influenced the interpretation of direct and moderating relationships with other domains in our hypothesized framework. Therefore, future studies might benefit from including other experience attributes in order to get insights into the possible associations with other variables and domains. Another limitation is that subjective well-being was utilized as a key concept of passengers’ behavioral variables. The theoretical framework should be expanded in future investigations by including more meaningful indicators of consumer behavior that reflect the decision-making process.

## 6. Conclusions

The current study included a sample of airline passengers to assess the role of the green physical environment at King Fahd International Airport on enhancing passengers’ experience and well-being and the engagement with the airport brand. Based on a validated structural model, the current study showed that the eco-design positively influenced passengers’ well-being and engagement with the brand. The subjective well-being was also influenced by passengers’ experience and brand engagement. The current findings also showed no significant moderating role of brand experience on the relationship between eco-design and well-being. Our results support the arguments that a green physical environment positively affects the active engagement of passengers with an airport brand and customer well-being, and we suggest these important ingredients of airport passenger behavior are variables that warrant future emphasis by researchers and stakeholders in the airline industry. Airport managers are advised to implement green environmental measures and support sustainable, environment-friendly objects inside the airport system and in airport buildings, in order to directly enhance brand engagement and well-being and indirectly support brand experience.

## Figures and Tables

**Table 1:** Demographic characteristics of the participants.

Parameter	Category	N (%)
Gender	Male	113 (32.1%)
	Female	239 (67.9%)
Age	20–29	158 (44.9%)
	30–39	85 (24.1%)
	40–49	42 (11.9%)
	50 and above	67 (19.0%)
Marital status	Single	115 (32.7%)
	Married	183 (52.0%)
	Other	54 (15.3%)
Child	None	153 (43.5%)
	1 to 3	165 (46.9%)
	4 or more	34 (9.7%)
Education	High School	43 (12.2%)
	University	183 (52.0%)
	Graduate school and above	92 (26.1%)
	Other	34 (9.7%)
Monthly income (SAR)	1000–3999	85 (24.1%)
	4000–7999	51 (14.5%)
	8000–11,999	93 (26.4%)
	Above 12,000	123 (34.9%)

**Table 2:** Outcomes of the convergent validity.

Parameter/Item	SFL	VIF	AVE	Cα	CR	RhoA
Eco-design of airports			0.714	0.920	0.920	0.922
Eco_01	0.808	2.302				
Eco_02	0.843	2.616				
Eco_03	0.882	3.101				
Eco_04	0.854	2.691				
Eco_05	0.819	2.292				
Eco_06	0.861	2.720				
Brand engagement			0.731	0.947	0.948	0.949
Eng_01	0.819	2.765				
Eng_02	0.844	3.326				
Eng_03	0.819	2.753				
Eng_04	0.896	3.936				
Eng_05	0.864	3.336				
Eng_06	0.878	3.754				
Eng_07	0.884	3.782				
Eng_08	0.830	2.815				
Subjective well-being			0.789	0.735	0.740	0.750
Well_01	0.908	1.510				
Well_02	0.868	1.510				
Eco-design × Brand experience			0.732	0.921	0.845	0.750
Eco_01 × Exp	0.891	2.274				
Eco_02 × Exp	0.554	2.369				
Eco_03 × Exp	0.910	3.282				
Eco_04 × Exp	0.749	2.747				
Eco_05 × Exp	1.025	2.675				
Eco_06 × Exp	0.921	3.321				

CR: Composite reliability; Cα: Cronbach’s alpha; SFL: standardized factor loading; AVE: average variance extracted; VIF: variance inflation factor.

**Table 3:** Outcomes of the Fornell–Larcker (F-L) criteria.

Parameter	1	2	3	4	5
1. Eco-design	0.845				
2. Brand engagement	0.822	0.855			
3. Brand experience	0.67	0.786	1		
4. Eco-design × Brand experience	−0.102	−0.137	−0.058	0.855	
5. Well-being	0.553	0.564	0.504	−0.091	0.888

The square roots of average variance extracted values are list on the diagonal, whereas other values represented the correlation between different domains.

**Table 4:** Outcomes of the heterotrait–monotrait (HTMT) ratio of correlations.

Parameter	1	2	3	4	5
1. Eco-design	NA				
2. Brand engagement	0.849	NA			
3. Brand experience	0.696	0.805	NA		
4. Eco-design × Brand experience	0.094	0.116	0.065	NA	
5. Well-being	0.666	0.668	0.587	0.069	NA

The square roots of average variance extracted values are list on the diagonal, whereas other values represented the correlation between different domains. NA: non-applicable.

**Table 5:** Outcomes of the structural model.

Parameter	T Value	β	95%CI	*p*-Value
Eco → Eng (H1)	37.067	0.822	0.778 to 0.863	<0.0001
Eco → Exp (H2)	0.933	0.075	−0.078 to 0.240	0.176
Eco → Well (H3)	3.058	0.267	0.089 to 0.432	0.001
Eng → Exp (H4)	9.805	0.724	0.581 to 0.866	<0.0001
Eng → Well (H5)	2.046	0.226	0.015 to 0.446	0.021
Exp → Well (H6)	1.765	0.146	0.015 to 0.310	0.039
Eco × Exp → Well (H7)	−0.283	−0.021	−0.107 to 0.152	0.612

Eco: eco-design; Eng: brand engagement; Exp: brand experience; Well: well-being; CI: confidence interval.

## Data Availability

Data are available on request due to privacy/ethical restrictions.

## Supplementary Materials

The following supporting information can be downloaded at: https://www.mdpi.com/article/10.3390/ijerph20020938/s1, Table S1: The originally developed questionnaire items; Table S2: Results of the bootstrap heterotrait–monotrait (HTMT) ratio of correlations.
