# Supplementary material for: Travelers’ Subjective Well-Being as an Environmental Practice: Do Airport Buildings’ Eco-Design, Brand Engagement, and Brand Experience Matter?
Source: Int J Environ Res Public Health. 2023 Jan 4;20(2):938. doi: 10.3390/ijerph20020938 (PMC9859295; doi:10.3390/ijerph20020938)
Supplement: Supplementary file 1 [file ijerph-20-00938-s001.zip › ijerph-2069551-supplementary.pdf]

Table S1. The originally developed questionnaire items.

Domain	Item Code	Item
Demographic characteristics	Dem_01	Gender
	Dem_02	Age
	Dem_03	Marital status
	Dem_04	Child
	Dem_05	Education
	Dem_06	Monthly income
Eco-design of airports	Eco_01	Eco spaces are readily available at this airport (ECO SPACES)
	Eco_02	Rest areas at this airport are designed in a green way (GREEN REST AREAS)
	Eco_03	Diverse flowers/trees and potted plants are located in many places (e.g., cafés, restaurants, shopping places) at this airport (PLANTS)
	Eco_04	A variety of green interior decorations are easily observable at this airport ECO FRIENDLY DÉCOR)
	Eco_05	Natural light through glass windows, walls, and roofs are easily observable at this airport (natural light)
	Eco_06	Air quality at this airport (e.g., temperature, circulation, humidity, natural scent, and ventilation) is fresh and comfortable (air freshness).
	Eco_07	Overall physical environment of this airport is designed in an eco-friendly way (eco-friendly physical environment).
Brand engagement	Eng_01	I have posted pictures/graphics related to this airport
	Eng_02	I have shared/commented on the airport related posts
	Eng_03	I have said positive things about this airport and its eco-design to others
	Eng_04	I will encourage friends and relatives to use airport in the future
	Eng_05	I will make constructive suggestions to the airport about how to improve its eco design
	Eng_06	I will let the airport know of ways that can better serve my needs to eco
	Eng_07	I would contact the airport if its eco were continually below expectations
	Eng_08	I would post negative comments if the airport's eco continued to be below expectations
Brand experience	Exp_01	I find the eco-design interesting in a sensory way
Subjective well-being	Well_01	I feel healthy and happy when staying at this airport
	Well_02	I feel emotional well-being while staying at this airport

Table S2. Results of the bootstrap heterotrait-monotrait (HTMT) ratio of correlations.

**Parameter**	**T stat**	**Bootstrap Mean**	**95% CI**
Eco $\rightarrow$ Eng	39.46	0.88	0.84 to 0.91
Eco $\rightarrow$ Exp	17.78	0.70	0.63 to 0.76
Eco $\rightarrow$ Eco × Exp	2.86	0.12	0.08 to 0.18
Eco $\rightarrow$ Well	12.15	0.67	0.58 to 0.76
Eng $\rightarrow$ Exp	30.46	0.80	0.76 to 0.85
Eng $\rightarrow$ Eco × Exp	2.55	0.14	0.08 to 0.22
Eng $\rightarrow$ Well	12.93	0.67	0.58 to 0.75
Exp $\rightarrow$ Eco × Exp	1.77	0.09	0.04 to 0.17
Exp $\rightarrow$ Well	10.96	0.59	0.49 to 0.67
Eco × Exp $\rightarrow$ Well	2.20	0.10	0.05 to 0.15
